# Educational disparities in adult health across U.S. states: Larger disparities reflect economic factors

**DOI:** 10.3389/fpubh.2022.966434

**Published:** 2022-08-16

**Authors:** Jennifer Karas Montez, Kent Jason Cheng

**Affiliations:** ^1^Department of Sociology, Syracuse University, Syracuse, NY, United States; ^2^Department of Social Science, Syracuse University, Syracuse, NY, United States

**Keywords:** education, health, disparities, fundamental cause, U.S. states

## Abstract

**Introduction:**

Education level is positively associated with adult health in the United States. However, new research shows that the association is stronger in some U.S. states than others, and that states with stronger associations also tend to have poorer overall levels of health. Understanding why educational disparities in health are larger in some states than others can advance knowledge of the major drivers of these disparities, between individuals and states. To that end, this study examined how key mechanisms (economic conditions, health behaviors, family, healthcare) help explain the education-health association in each state and whether they do so systematically.

**Methods:**

Using data on over 1.7 million adults ages 25–64 in the 2011–2018 Behavioral Risk Factor Surveillance System, we estimated the association between education level and self-rated health in each state, net of age, sex, race/ethnicity, and calendar year. We then estimated the contribution of economic, behavioral, family, and healthcare mechanisms to the association in each state.

**Results:**

The strength of the education-health association differed markedly across states and was strongest in the Midwest and South. Collectively, the mechanisms accounted for most of the association in all states, from 55% of it in North Dakota to 73% in Oklahoma. Economic (employment, income) and behavioral (smoking, obesity) mechanisms were key, but their contribution to the association differed systematically across states. In states with stronger education-health associations, economic conditions were the dominant mechanism linking education to health, but in states with weaker associations, the contribution of economic mechanisms waned and that of behavioral mechanisms rose.

**Discussion:**

Meaningful reductions in educational disparities in health, and overall improvements in health, may come from prioritizing access to employment and livable income among adults without a 4-year college degree, particularly in Southern and Midwestern states.

## Introduction

An adult's education level is a robust predictor of their health in the United States. Compared to their less-educated peers, adults with more education have better overall health, are less likely to develop morbidities and disability, and tend to live longer and spend more of those years in good health (1, 2). The magnitude of these disparities is striking. Among U.S. adults in their mid-40s, <15% of those without a high school diploma reported being in excellent health, compared to 24% of those with a high school diploma, over 40% of those with a 4-year college degree, and over 50% of those with a doctorate or professional degree (3). In recent decades, disparities in health between adults with and without a 4-year college degree have become especially pronounced (4, 5).

**Figure 1:**
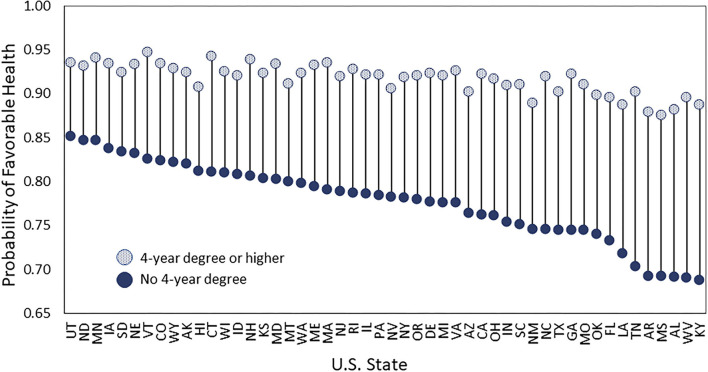
Probability of reporting favorable health among adults ages 25–64 by U.S. state. Data are from the 2011–2018 BRFSS, include adults ages 25–64, and are adjusted for age, sex, and race-ethnicity differences between states. Adults who reported that their health was excellent, very good, or good are considered in “favorable” health, unlike those who reported that their health was fair or poor.

New research finds that the importance of one's education level for health differs markedly across U.S. states (6–8). As an example, Figure 1 shows the association between education and self-rated health by state among adults ages 25–64 (the associations are adjusted for age, sex, and race-ethnicity differences across states' populations). The association is strongest in West Virginia, where just 69% of adults without a 4-year college degree report being in favorable health compared to 90% of their more-educated peers, a gap of 21 percentage points. The association is weakest in Utah, where 85% of adults without a 4-year degree and 94% of their more-educated peers are in favorable health, a gap of just 9 percentage points. Also intriguing, this new area of research finds that states with the largest disparities in health across education levels tend to have the worst overall health (6–8). In other words, these states are especially disadvantaged. Taken together, these findings imply that understanding why education is a stronger predictor of health in some states than others could advance knowledge of the major drivers of health levels and disparities, between individuals and between states.

A key framework for understanding the education-health association is Fundamental Cause Theory (FCT). It asserts that education is important in contexts with the resources to avoid disease and premature death, yet more-educated persons have greater access to those resources (9–11). Indeed, compared to their less-educated peers, more-educated U.S. adults have greater access to four types of salubrious resources: economic well-being, social ties, healthy behaviors, and quality health care (2, 12). Those four types of resources, or “mechanisms,” help explain a large share of the education-health association in the country today (2, 12). Central to the current study, the relevance of each mechanism for explaining the education-health association may vary across states. For instance, unemployment rates, the share of jobs requiring a college degree, and median income vary across states (13). Access to affordable health care for disadvantaged adults also varies across states. Having low education may pose substantial barriers to health care in states that offer minimal levels of Medicaid benefits. As another example, the relevance of smoking for the education-health association may partly depend on states' tobacco control policies (6). If the salience of such mechanisms differs across states, this information may point to reasons why the education-health association is stronger in some states than others (i.e., certain mechanisms may be key) or it may have no bearing on the strength of the association (i.e., a high salience of one mechanism in a state may simply be offset by the low salience of another).

This study examines how the importance of four key mechanisms (economic conditions, health behaviors, social factors, healthcare) linking educational attainment to self-rated health differs across states. Using data spanning 2011–2018 from over 1.7 million U.S. adults ages 25–64 years, it assesses how much of the education-health association in each state is accounted for by these mechanisms, and whether their importance differs across states in a systematic way. In other words, does the importance of these mechanisms vary across states? Such patterns can provide insights into why the education-health association is stronger in some states than others and point to strategies to reduce educational disparities in health and improve overall health.

## Materials and methods

### Data and sample

We used data from the Behavioral Risk Factor Surveillance System (BRFSS), an annual cross-sectional survey of the noninstitutionalized U.S. adult population aged 18 and older. The BRFSS is the best available data to examine the education-health association within states because the dataset is large, representative of noninstitutionalized adults at the state level, and contains information on educational attainment, health, and the four mechanisms examined in this study.

We used BRFSS data from 2011 through 2018. We start in 2011 when BRFSS expanded the sample to also include households with only cell phones and revised the weighting methodology (14). We restricted the sample to adults ages 25–64 years. The lower age limit was set at 25 because our main exposure is completed education through a Bachelors' degree. The upper limit was set at 64 because some of the mechanisms, such as employment, are most relevant for working ages. The 2011–2018 BRFSS contains 2,172,540 adults ages 25–64 years.

### Self-rated health and educational attainment

We examine self-rated health, a valid indicator of overall health (15). BRFSS asks adults, “Would you say that in general your health is excellent, very good, good, fair, or poor?” We dichotomized the responses, as typically done, so that 1 = excellent, very good, or good (which we refer to as “favorable health”) and 0 = fair or poor. The dichotomization is advantageous for the present study because it avoids a complication that would arise from using all responses in ordinal logit models, as the proportional odds assumption is likely to be violated in some U.S. states but not others.

To capture educational attainment, BRFSS asks respondents, “What is the highest grade or year of school you completed?” It has six response categories: never attended school or kindergarten, grades 1–8, grades 9–11, grade 12 or GED, 1–3 years of college, and four or more years of college. Our preferred specification is a dichotomous indicator, where 1 = four or more years of college (we call this group college graduates). It reflects studies showing that the health of U.S. adults has bifurcated, with college graduates doing well and others doing poorly (5, 16, 17) and that health disparities between states are largest for those without a 4-year college degree (6, 7).

### Hypothesized mechanisms

We examined four types of mechanisms: economic conditions, health behaviors, social factors, and healthcare. We refer to them as mechanisms because they are hypothesized to be key pathways linking education to health (2, 12) and because this term is prominently used in FCT (10). Although the term “mechanism” often has a causal connotation, we make no causal claims in this analysis.

All mechanisms were measured as continuous, ordinal, or binary variables to facilitate the mediation analysis described below. The two economic factors were employment status (1 = currently employed) and annual household income. To obtain information on employment status, BRFSS provides eight possible employment categories (e.g., employed for wages, self-employed, out of work for 1 year or more) and asks respondents to select the category that best describes them currently. The BRFSS asks respondents about their annual income from all sources. It provides the responses in categories of varying widths (< $10,000; $10,000 to < $15,000;…;$50,000 to < $75,000; and ≥ $75,000). We converted this measure into a continuous one based on the recommendation of a validation study, which found that using the upper limit of each category provided the best overall match to the actual income distribution (18).

Three behavior-related mechanisms included smoking, heavy drinking, and obesity. BRFSS assigns a smoking status to respondents based on their answers to questions about past and current cigarette smoking. We included a binary indicator of smoking status, with never smoker = 1 and current and former smoker = 0. The survey provides a binary indicator of heavy drinking (defined as more than 14 drinks per week among men and more than 7 drinks per week among women) based on respondents' answers to questions about the frequency and quantity of alcoholic beverage consumption during the past 30 days. As a proxy for health-related behaviors, we also included a measure of obesity, defined as a body mass index of 30 or higher. The BRFSS calculates BMI based on respondents' reports of their height and weight without shoes.

For the social mechanisms, we included two measures of family, given that family composition differs across education levels and is considered one of the key social mechanisms liking education to health (12). The BRFSS does not contain measures of other social factors such as friendships or loneliness. Specifically, we included the self-reported number of children under 18 years of age in the household (top coded at 10) and self-reported marital status (1 = married). Lastly, the analysis incorporated two healthcare mechanisms related to healthcare availability and affordability. The availability question asked adults if they currently had “any kind of health care coverage, including health insurance, prepaid plans such as HMOs, or government plans such as Medicare, or Indian Health Service.” The affordability question asked, “was there a time in the past 12 months when you needed to see a doctor but could not because of cost?”

### Covariates

We included calendar year and three self-reported covariates, age, sex, and race/ethnicity. We accounted for age, sex, and race/ethnicity because they are related to both educational attainment and health and because their relevance for the education-health association may differ across states. For instance, a recent study showed that higher education does not provide the same degree of cardiometabolic health benefits for Black adults as it does for White or Hispanic adults (19). We measured age in 5-year groups, from 25 to 29 through 60–64 years. The BRFSS provides sex as female or male. The BRFSS combines respondents' answers to a question about Hispanic/Latinx/Spanish origin and a question about which group (White; Black or African American; American Indian or Alaskan Native; Asian; Pacific Islander) best represents their race into a single variable identifying respondents as non-Hispanic Black, non-Hispanic Other, non-Hispanic White, and Hispanic.

### Methods

We estimated logistic regression models using the form below, where *b*_1_ is the coefficient of interest. The vectors *b*_2_*, b*_3_*, b*_4_, and *b*_5_ represent the coefficients for the economic, behavioral, family, and healthcare mechanisms, respectively. The *b*_6_ vector contains coefficients for the covariates, age, sex, race-ethnicity, and calendar year.


$$\begin{array}{l} \ln\left(\frac{p}{\left(1-p\right)}\right)=b_{0}+b_{1}college+b_{2}economic+b_{3}behaviors\\ \;+b_{4}family+b_{5}healthcare+\;b_{6}covariates \end{array}$$

We estimated a model for each state, which achieves the aims of the study because it allows the importance of the mechanisms in accounting for the education-health association to differ across states. Alternatively, achieving these aims with one model containing all 50 states would require interactions between each state and education and the nine mechanisms (i.e., nearly 500 interaction terms). The notional simplicity of a one-model approach is outweighed by the complexity of hundreds of interaction terms in the mediation analysis.

To examine the contribution of the hypothesized mechanisms to the education-health association within states, we used the method developed by Karlson, Holm, and Breen (KHB) to assess mediation in non-linear probability models (20). It decomposes the difference in the logit coefficient of a variable *X* (in our case, *college*'s coefficient, *b*_1_) between models with and without the mechanisms *Z* (i.e., economic, behavioral, family, healthcare), into the portion attributable to *Z*, while accounting for the rescaling of the *X* coefficient that occurs across nested non-linear probability models.

A few respondents were missing information on some variables. In preliminary analyses, we assessed several approaches for handling the missing information, such as excluding respondents with missing data or using multiple imputation (details are in Supplementary Tables 1, 2). Because the findings were similar for both approaches, we chose the former one for our main analyses, which includes 1,716,757 adults. All models were estimated with Stata MP 17.

## Results

We first describe a few key descriptive statistics from Table 1. Among U.S. adults ages 25–64 during 2011–2018, 83% reported being in favorable health. This percentage ranged from 76% in West Virginia to 89% in Minnesota and Vermont. The percentage of college graduates ranged from 21% in West Virginia to 45% in Massachusetts. States differed in several of the mechanisms. For example, the percentage of adults who had never smoked ranged from 45% in Kentucky and West Virginia to 72% in Utah, and percentage of those who were employed ranged from 63% in West Virginia to 82% in North and South Dakota. In contrast, some mechanisms differed little across states, such as the prevalence of heavy alcohol consumption and the number of children in the household.

**Table 1:** Weighted descriptive statistics of U.S. adults ages 25–64 by state.

	**Favorable health**	**College graduate**	**Employed**	**Household income**	**Never smoked**	**Heavy drinker**	**Obese**	**Married**	**Number of children in the home**	**Healthcare coverage**	**Healthcare not affordable**
AL	78	25	64	49,321	52	6	39	61	0.85	83	21
AK	86	29	74	58,956	51	8	32	65	0.99	85	15
AZ	82	29	68	51,637	57	7	32	63	1.04	83	18
AR	77	22	65	46,983	48	6	39	63	0.94	82	21
CA	82	32	70	52,866	64	7	28	64	0.97	84	16
CO	87	40	76	58,150	57	7	24	67	0.92	86	15
CT	88	40	77	59,708	57	7	29	65	0.83	91	12
DE	85	31	75	55,882	54	7	34	61	0.89	89	14
FL	82	29	69	50,674	55	8	31	60	0.83	79	22
GA	82	30	69	51,158	58	6	34	61	0.92	79	21
HI	86	32	78	56,737	58	9	27	62	0.91	92	9
ID	86	27	73	53,558	60	7	31	71	1.17	81	18
IL	84	34	73	55,217	57	7	33	63	0.91	86	14
IN	82	26	72	53,240	50	6	36	65	0.97	85	17
IA	88	30	80	57,719	54	8	36	70	1.00	91	10
KS	85	34	76	55,967	55	6	36	69	1.00	85	15
KY	78	24	66	51,360	45	7	38	63	0.87	87	18
LA	79	24	67	49,896	52	7	39	57	0.93	81	21
ME	85	29	74	54,107	47	9	32	67	0.76	87	13
MD	87	40	77	59,822	61	6	33	62	0.87	90	12
MA	88	45	77	60,140	57	8	26	64	0.80	94	10
MI	83	29	69	53,815	50	8	35	63	0.89	88	16
MN	89	37	81	59,970	55	8	30	68	0.95	91	11
MS	77	22	65	45,213	53	6	41	56	0.91	78	24
MO	83	30	72	53,953	50	8	35	65	0.91	85	16
MT	85	31	74	52,752	53	9	28	67	0.90	83	15
NE	87	32	80	56,780	56	8	35	69	1.03	86	14
NV	82	24	70	52,169	56	7	29	61	0.97	79	19
NH	88	37	77	60,749	52	8	30	69	0.81	89	12
NJ	85	40	75	59,052	59	5	29	65	0.88	87	15
NM	80	26	67	46,997	56	6	32	60	0.99	83	19
NY	85	37	72	53,866	59	6	28	60	0.86	88	14
NC	82	31	71	52,257	54	6	35	63	0.82	82	19
ND	88	31	82	59,903	53	8	36	69	0.93	90	9
OH	83	28	72	53,784	50	7	35	63	0.90	89	14
OK	80	26	69	51,190	51	5	37	65	0.96	82	19
OR	84	33	69	53,885	55	9	31	66	0.84	86	17
PA	84	32	73	56,104	52	7	33	63	0.85	89	14
RI	85	34	73	55,822	54	7	30	62	0.80	89	14
SC	82	27	70	50,348	52	7	37	61	0.87	81	20
SD	88	30	82	56,686	52	7	33	69	0.99	89	12
TN	79	26	68	49,686	51	5	37	62	0.85	83	19
TX	82	29	72	52,011	61	8	36	65	1.04	74	21
UT	88	33	76	59,690	72	5	28	74	1.43	86	15
VT	89	37	79	56,878	52	9	28	66	0.75	91	10
VA	85	39	76	57,941	57	6	32	65	0.88	87	15
WA	86	34	72	57,661	57	8	30	67	0.87	87	14
WV	76	21	63	48,091	45	4	40	64	0.80	85	20
WI	86	30	77	56,440	53	9	33	66	0.90	90	13
WY	87	27	76	57,528	53	7	31	68	0.96	82	16
Min	76	21	63	45,213	45	4	24	56	0.75	74	9
U.S.	83	31	72	53,897	56	7	32	63	0.92	84	17
Max	89	45	82	60,749	72	9	41	74	1.43	94	24

*All numbers are percentages, except for household income ($) and number of children in the home (N=1,716,757)*.

We then estimated the 50 state-specific logistic regression models predicting favorable health. In all 50 states, having a college degree is associated with a significantly higher probability of reporting favorable health but the magnitude of the association differs across states, consistent with prior research and Figure 1. Supplementary Figure 1 displays the college coefficient estimated from each of the 50 models along with 95% confidence intervals (it shows that all 50 coefficients are significantly different from zero). Supplementary Figure 2 adjusts the confidence intervals so that comparisons between states can be made (it shows significant differences between many states). We used the 50 state-specific regression models to answer our two research questions, as described below.

### How do the mechanisms contribute to the education-health association across states?

**Figure 2:**
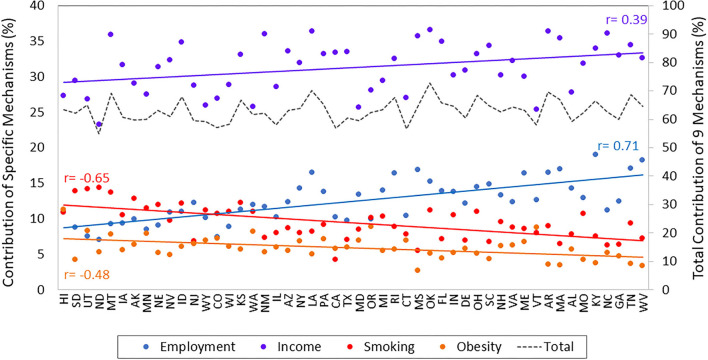
Contribution of four key mechanisms to the education-health association in U.S. states. Data are from the 2011–2018 BRFSS and include adults ages 25–64 years. States are ordered from left to right in ascending order of the strength of their education-health association. *r* = correlation between the strength of the education-health association in each state and the percentage contribution of each mechanism.

Figure 2 shows how much of the education-health association in each state is explained by the nine mechanisms in total (detailed model results are in Supplementary Table 3). The total contribution of the mechanisms, shown as the dashed gray line, differs considerably across states. They explain as little as 55% of the education-health association in North Dakota and as much as 73% in Oklahoma, an 18 percentage-point difference. To explore systematic patterns, Figure 2 shows states sorted from left to right in ascending order of the strength of the education-health association. As a group, the mechanisms are not much better at explaining stronger or weaker associations. This is evidenced by the relatively horizontal dashed gray line and weak correlation (*r* = 0.23, *p* = 0.11) between the total contribution of the mechanisms and strength of the education-health association across the states.

### How does the importance of each mechanism vary across states? Do mechanisms vary?

**Figure 3:**
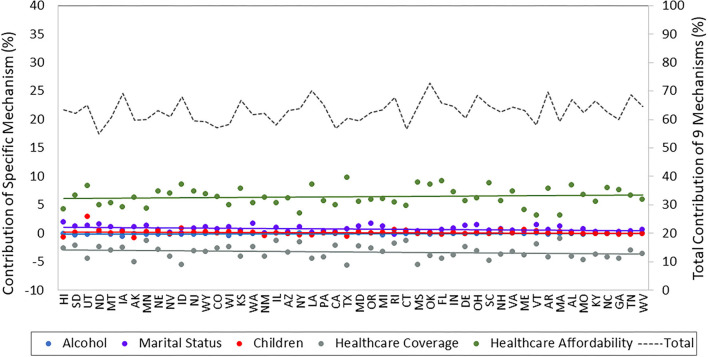
Contribution of other mechanisms to the education-health association in U.S. states. Data are from the 2011–2018 BRFSS and include adults ages 25–64 years. States are ordered from left to right in ascending order of the strength of their education-health association.

Using the same models from above, we examine the contribution of each of the nine mechanisms to the education-health association within states (i.e., the sum of the contribution of the nine mechanisms = the total contribution). The contributions of income, employment, smoking, and obesity were large and unequal across states, as shown in Figure 2 (the contributions of the other five mechanisms were small and differed little across states, as shown in Figure 3). Figure 2 reveals several intriguing patterns. First, income is the dominant contributor across all 50 states. Nevertheless, income's contribution varies considerably from just 23% in North Dakota to 37% in Oklahoma. Second, the contributions of both income and employment rise across states as the association becomes stronger (i.e., their contribution is larger in states on the right side of Figure 2 than for those on the left side). We can quantify this pattern: the correlation between the strength of the education-health association and the contribution of income is 0.39 (*p* < 0.01) and the correlation between the strength of the education-health association and the contribution of employment is 0.71 (*p* < 0.001). Third, in contrast to economic mechanisms, the contribution of behavior-related mechanisms decreases across states as the education-health association becomes stronger. Consequently, in states like West Virginia and Tennessee with strong education-health associations, the contribution of employment (17.7%) is more than double that of smoking (8.3%), while in states like Hawaii and South Dakota with weak associations, smoking (12.5%) contributes more than employment (9.9%). Taken together, these three patterns are consistent with the notion of mechanisms changing from context to context in the FCT.

**Figure 4:**
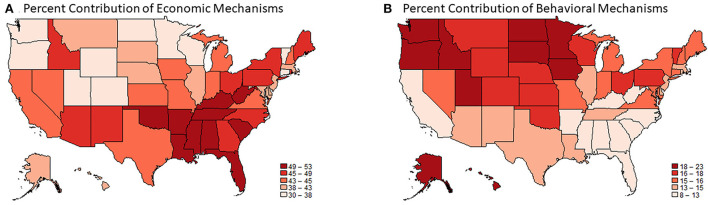
The contribution of economic **(A)** and behavioral **(B)** mechanisms to the education-health association in U.S. states. Data are from the 2011–2018 Behavioral Risk Factor Surveillance System and include adults ages 25–64. The magnitude of the education-health association is the difference in the probably of favorable health among adults with at least a 4-year college degree minus the probability of favorable health among adults without a 4-year college degree. The probabilities are estimated from state-specific logistic regression models which include an indicator for college degree, age, sex, race-ethnicity, and calendar year.

Another view of how mechanisms vary across states is provided in Figure 4. Panel A shades states according to the contribution of the economic mechanisms (income, employment) to the association, where darker shades of red indicate a larger contribution. Panel B shades states according to the contribution of the behavioral mechanisms (smoking, obesity) to the associations. The two panels are near mirror images of each other: states where economic mechanisms are particularly important in explaining the education-health association, such as states in the South and Appalachia, tend to be the same states where behavior-related mechanisms are least important.

### Robustness checks

We replicated the analyses using different regression models and specifications of education and self-rated health. We first assessed whether our findings were consistent when using a linear probability model instead of a logistic regression model. The findings from these analyses (Supplementary Figure 3) corroborate our main findings. That is, the contribution of economic and behavioral mechanisms to the education-health association were sizable but differed systematically across states such that the importance of economic mechanisms was higher, where the relevance behavioral mechanisms were lower, as the education-health association increased. For example, in the state with the strongest education-health association, income and employment accounted for 48% of it, while smoking and obesity accounted for 11%; in the state with the weakest association, income and employment accounted for 28% and smoking and obesity accounted for 20%. In the linear probability models, the importance of employment rivaled that of income in many Midwestern and Southern states where the education-health association was strongest (e.g., WV, TN, KY, AR, AL, MS, GA).

Next, we assessed whether our findings were consistent when using a different specification of education level. Recall that the BRFSS provides a 6-category measure of education: never attended school or kindergarten, grades 1–8, grades 9–11, grade 12 or GED, 1–3 years of college, and four or more years of college. We created a pseudo-continuous measure by imputing approximate years of schooling (0, 4.5, 10, 12, 14, 18 years) to each category. Although this measure uses all information in the BRFSS, it has only six possible values, thereby posing challenges to estimating a linear relationship with self-rated health. Nevertheless, we replicated the analyses using this measure with both the logistic and linear probability models. The overall findings were similar to those when using the binary measure. That is, the contribution of economic and behavioral mechanisms was large but differed across states such that when the importance of economic mechanisms was higher, that of behavioral mechanisms was lower, as the education-health association became stronger (Supplementary Figures 4, 5). Among three of the four analyses (two regression models x two measures of education), income generally contributed more than employment to the association. The exception was the logistic model with the pseudo-continuous measure where employment generally contributed more than income. Unsurprisingly, given the drawback of the pseudo-continuous measure mentioned previously, the mechanisms did not explain as much of the education-health association with the pseudo-continuous measure as they did with the binary measure. Using logistic regression, the mechanisms collectively explained 55 to 73% of the education-health association in each state when using the binary measure and 34 to 55% when using the pseudo-continuous measure. Lastly, our results were robust to using an OLS with all five values of self-rated health (Supplementary Figure 6).

## Discussion

New research has shown that the association between educational level and health is stronger in some U.S. states than others, and that states with stronger associations also tend to have poorer overall levels of health. Understanding why educational disparities in health are larger in some states than others can advance knowledge of the major drivers of these disparities, between individuals and states. To that end, this study examined how key mechanisms—economic conditions, health behaviors, family, and healthcare—help explain the education-health association in each state and if they do so systematically. Below, we summarize four key findings.

The first set of findings is descriptive. Specifically, the strength of the association between education level and self-reported health differed markedly across U.S. states. It differed across states mainly because the health of nongraduates differed, consistent with prior work (6, 7). For instance, as shown in Figure 1, 90% of adults in West Virginia with at least a 4-year degree were in favorable health, as were 94% of their peers in Utah, while 69% of adults in West Virginia without a 4-year degree were in favorable health, as were 85% of their peers in Utah. This pattern comports with the notion that higher education acts as a “personal firewall” to protect health across contexts (7). Nevertheless, there appears to be limits on how much protection a college degree affords in contexts that are highly problematic for health. For instance, in many states in the South and Midwest (e.g., KY, WV, AL, MI, AR) where nongraduates had strikingly worse health than the rest of the country, the health of graduates also suffered considerably. Such states are especially disadvantaged: they have large disparities in health across education levels and relatively low overall levels of health.

Second, in states where the education-health association was especially strong, economic conditions were the dominant mechanism linking education to health. This suggests that educational disparities in health are exacerbated when less-educated adults have especially limited access to employment and income needed for health-sustaining resources such as nutritious food and safe housing. To the extent that these states improved opportunities for desirable employment and livable wages among college nongraduates, the largest educational disparities in health in the country may be substantially reduced. In other words, states that provide opportunities for economic well-being among nongraduates—for example, through higher minimum wage, earned income tax credits, worker protections, and robust labor markets—may be able to disrupt the pathway from education to economic conditions to health. One interpretation is that structural factors, particularly labor markets, are central for explaining the largest educational disparities in health in the country.

A third key finding is that in states where the link between education level and economic conditions was not as strong, health-related behaviors were more relevant in explaining the education-health association. Specifically, looking across states, as the association became weaker, the contribution of economic mechanisms to the association fell while that of behavior-related mechanisms rose. In states with the weakest associations, smoking rivaled employment as the second most important contributor (income was generally the most important). This pattern would have been obscured if we had only examined the total contribution of the mechanisms, as the total did not rise or fall across states according to the strength of the association. In other words, the importance of certain mechanisms varies from context to context, consistent with a core premise of FCT. Even though the importance of the mechanisms varies across contexts, our findings point to improving employment and income among nongraduates as a potentially effective strategy, as the largest disparities are in states where economic conditions are the dominant mechanism linking education to health.

Fourth, the mechanisms often hypothesized to explain the education-health association (economic conditions, behaviors, social factors such as family, and healthcare) were better able to explain the association in some states than others. Collectively, the total contribution of the mechanisms accounted for as little as 55% of the association in North Dakota to as much as 73% in Oklahoma, a range of 18 percentage points. This range largely reflects the varying contribution of economic conditions. In general, the more closely that education was tied to economic conditions in a state, the more of association that we explained. Among the 10 states where we were best able to explain the association, economic conditions were the single dominant mechanism in some (LA, AR, TN, AL, RI) and shared a high degree of importance with behaviors in others (ID, IA, KS, OK, OH). Among the 10 states where we were least able to explain the association (ND, CT, CA, CO, IL, VT, WI, WY, MA, MD), factors other than those examined in this study also carry considerable weight.

Our findings generally comport with FCT. The fact that college graduates had better health than nongraduates in all 50 states supports FCT's assertion that educational disparities in health persist because more-educated adults use their resources to secure health advantages across contexts. In addition, our finding that nongraduates' health differed markedly across states aligns with FCT's claim that it is essential to understand what puts lower SES individuals “at risks of risks.” In states where the education-health association was strongest, less-educated adults were at particularly high risk of adverse economic conditions. Also consistent with FCT, we find evidence of mechanisms varying across places, such that education-health association exists across all 50 states even though the mechanisms that help explain the association vary in importance across states.

### Implications for reducing health disparities between states and individuals

Our findings suggest that strategies to weaken educational disparities in health, and improve overall levels of health, might benefit by incorporating both national and state-level elements. Income may be one of the national elements. Regardless of states' political orientation, demographic composition, macroeconomic conditions, or any other characteristic, income was a central mechanism linking higher education to better health. This suggests that improving opportunities for higher incomes (e.g., raising the federal minimum wage to keep up with inflation) among college nongraduates may be a first-order strategy for reducing health inequalities. Also relevant is our finding that the largest educational disparities in health are in the Midwest and South, where nongraduates are especially disadvantaged in income and employment. This suggests that the largest reductions in educational disparities in health may come from prioritizing improvements in economic conditions in these parts of the country. Our findings also suggest that certain state-specific strategies may be beneficial. For example, in states like Washington and Minnesota, a focus on health behaviors may be key, while in states like Ohio and Pennsylvania, a two-pronged approach that focuses on both economic conditions and health behaviors may be required (as evident from Figure 4).

### Limitations and future research

Despite the strengths of the data and analysis, the study has some limitations. First, our data are cross-sectional and lack retrospective information about respondents' lives, so we cannot assert a temporal order between education, the mechanisms, and health. It is possible that the order is reversed for some respondents. For instance, unfavorable health in childhood can truncate schooling, an effect that may be most severe in states lacking educational supports and other compensatory resources. This may exacerbate the magnitude of educational disparities in health, as states where poor childhood health presents major obstacles to obtaining higher levels of education are likely to be the same states where higher levels of education are immensely important for obtaining health enhancing resources The lack of retrospective information also means that the mechanisms only reflect the time of survey. Having information such as employment and marital histories may have helped account for more of the education-health association. Moreover, our short time series did not allow us to examine temporal mechanism swapping. We were only able to assess how mechanisms varied across place, not across time. Second, our study was not designed to assess causality. We do not claim that education caused the mechanisms which, in turn, caused health. We rely on existing literature using causal methods [e.g., (21)] that identified effects of education on health-related outcomes to judiciously interpret our findings.

It is also important to consider that the BRFSS sampling frame excludes incarcerated persons. Because incarcerated adults tend to have relatively low levels of education and poorer health, the size of the education-health association in states with high incarceration rates, including many states in the South and Midwest (22), may be underestimated. Thus, our findings may be even more pronounced if the BRFSS contained incarcerated persons, because states with large educational disparities in health tend to be those with high incarceration rates. Incarceration may also operate as a mechanism through which low education results in poor health, given the many pernicious downstream consequences of current and former incarceration on employment, income, families, social ties, health, and more.

Another potential shortcoming is that our study lacked information on immigration and interstate migration. The proportion of a state's population who are immigrants could affect our findings, given that the education-health association is weaker for some immigrant groups and their health tends to be more favorable than US-born individuals. Supplementary analyses provide some assurance that our findings are not materially affected. Specifically, there is little correlation between the percentage of immigrants in a state and the strength of the state's education-health associations (*r* = −0.15, *p* = 0.30) and there is a small and non-significant correlation between the percentage of immigrants and the contribution of the two dominant mechanisms, employment and income, to the association. Interstate migration could potentially affect our findings to the extent that education or health influences interstate migration. Although we do not rule out this possibility, findings from other studies suggest that it does not materially alter our findings. For example, one study of the education-disability association across states showed the cross-state pattern persisted after limiting the sample to non-movers (7), another study concluded that interstate migration of less- or more- educated adults does not explain the growing health divides across states (23), and a third study found that the benefits of education for health were mainly shaped by their adulthood contexts, not their childhood contexts (24).

This study laid a foundation for a new line of research on why the salience of educational attainment for health differs across U.S. states. It borrowed an approach from decades of research on why the salience of education for health has grown over time (9), which has examined how the purported mechanisms linking education to health have been changing over time. Rather than examining how mechanisms change over time, we examined how they differed across place. Even though these previous studies and the current study examined mechanisms, the ultimate goal is to uncover clues about the structural level factors that made those mechanisms salient and have the potential to reduce health disparities. Given the prime role of employment and income in accounting for the largest disparities in Midwestern and Southern states that we identified, an important next step is to investigate the structural factors (e.g., states' minimum wage levels, earned income tax credits, paid leave laws) that lie at the root of the disparities and patterns that we documented. Ultimately, reducing the disparities will require identifying and addressing these structural factors.

It may also be informative to examine additional mechanisms, such as occupation, drug use, non-familial social relationships, exposure to discrimination, and lifetime exposures to the mechanisms. It may also be fruitful to examine how and why the education-health association differs across states for specific demographic subgroups (e.g., for gender and race/ethnic groups), and how and why the association differs across local areas. Our study is a first step toward a better understanding of how geographic contexts shapes the importance of one's education level for their health.

## Conclusions

Not having a 4-year college degree is much riskier for health in some U.S. states than in others. It is especially risky in Southern and Midwestern states. In general, these states have the largest educational disparities in health and the lowest overall levels of health: these states are especially disadvantaged. Meaningful reductions in educational disparities in health, and overall improvements in health, may come from prioritizing access to employment and livable income among adults without a 4-year college degree, particularly in Southern and Midwestern states.

## Data availability statement

Publicly available datasets were analyzed in this study. This data can be found at: www.cdc.gov/brfss/index.html.

## Author contributions

JKM developed the idea for the project and wrote the first draft of the manuscript. KJC conducted the statistical analyses, created the graphics, and edited the final version of the manuscript. Both authors contributed to the article and approved the submitted version.

## Funding

Research reported in this publication was supported by the National Institute on Aging of the National Institutes of Health under Award Numbers R01AG055481 and R24AG045061.

## Conflict of interest

The authors declare that the research was conducted in the absence of any commercial or financial relationships that could be construed as a potential conflict of interest.

## Publisher's note

All claims expressed in this article are solely those of the authors and do not necessarily represent those of their affiliated organizations, or those of the publisher, the editors and the reviewers. Any product that may be evaluated in this article, or claim that may be made by its manufacturer, is not guaranteed or endorsed by the publisher.

## Author disclaimer

The content is solely the responsibility of the authors and does not necessarily represent the official views of the National Institutes of Health.
