# Supplementary material for: Educational disparities in adult health across U.S. states: Larger disparities reflect economic factors
Source: Front Public Health. 2022 Aug 16;10:966434. doi: 10.3389/fpubh.2022.966434 (PMC9424624; doi:10.3389/fpubh.2022.966434)
Supplement: Supplementary file 1 [file Data_Sheet_1.docx]

Supplementary Material

| **Supplementary Table 1 \|** Missing data for each variable, 2011-2018 Behavioral Risk Factor Surveillance System | |
| --- | --- |
| Variables | Percentage of Total Respondents Missing Data  (N = 2,172,540) |
|  |  |
| Self-rated health | 0.3 |
| Educational Attainment | 0.3 |
| Mechanisms |  |
| Economic |  |
| *Employment* | 0.7 |
| *Income* | 11.3 |
| Behavioral |  |
| *Smoking* | 3.1 |
| *Drinking* | 5.7 |
| *Obesity* | 6.9 |
| Family |  |
| *Marital status* | 0.5 |
| *Children in home* | 0.7 |
| Healthcare |  |
| *Insurance Access* | 0.3 |
| *Affordability* | 0.3 |
| Controls |  |
| Sex | 0.0 |
| Race-ethnicity | 1.4 |
| Age | 0.0 |

| **Supplementary Table 2 \|** Education-health association among U.S. adults ages 25–64, 2011-2018; Comparing results using complete-case dataset to results using five multiply imputed datasets | | | | | | | | |
| --- | --- | --- | --- | --- | --- | --- | --- | --- |
|  | Complete-Case Dataset (N=1,716,757) | Multiply Imputed Datasets  (N= 2,165,628 in each) | | | | | | |
|  |  |  | 1 | 2 | 3 | 4 | 5 |  |
| OR^1^ for College Degree |  |  |  |  |  |  |  |  |
| …in reduced model | 3.52*** |  | 3.60*** | 3.60*** | 3.60*** | 3.60*** | 3.60*** |  |
| …in full model | 1.59*** |  | 1.62*** | 1.62*** | 1.62*** | 1.62*** | 1.62*** |  |
|  |  |  |  |  |  |  |  |  |
| Mediation (%) |  |  |  |  |  |  |  |  |
| **Total** | **63.12** |  | **62.52** | **62.50** | **62.50** | **62.57** | **62.43** |  |
|  |  |  |  |  |  |  |  |  |
| **Economic Conditions** | **44.14** |  | **44.17** | **44.20** | **44.20** | **44.21** | **44.14** |  |
| *Employment* | *12.48* |  | *12.56* | *12.54* | *12.54* | *12.55* | *12.54* |  |
| *Income* | *31.66* |  | *31.61* | *31.66* | *31.66* | *31.66* | *31.60* |  |
|  |  |  |  |  |  |  |  |  |
| **Health-related Behaviors** | **14.99** |  | **14.41** | **14.37** | **14.37** | **14.43** | **14.36** |  |
| *Smoking* | *8.89* |  | *8.54* | *8.50* | *8.51* | *8.53* | *8.50* |  |
| *Drinking* | *-0.09* |  | *-0.07* | *-0.07* | *-0.07* | *-0.08* | *-0.08* |  |
| *Obesity* | *6.19* |  | *5.94* | *5.94* | *5.93* | *5.98* | *5.94* |  |
|  |  |  |  |  |  |  |  |  |
| **Family Factors** | **0.67** |  | **0.78** | **0.79** | **0.78** | **0.78** | **0.78** |  |
| *Marital status* | *0.67* |  | *0.80* | *0.80* | *0.80* | *0.80* | *0.80* |  |
| *Children in home* | *0.00* |  | *-0.02* | *-0.01* | *-0.02* | *-0.02* | *-0.02* |  |
|  |  |  |  |  |  |  |  |  |
| **Healthcare** | **3.32** |  | **3.16** | **3.14** | **3.15** | **3.15** | **3.15** |  |
| *Insurance Access* | *-3.09* |  | *-3.06* | *-3.07* | *-3.06* | *-3.06* | *-3.06* |  |
| *Affordability* | *6.41* |  | *6.22* | *6.21* | *6.21* | *6.21* | *6.21* |  |
|  |  |  |  |  |  |  |  |  |

^1^ OR = Odds Ratio. The “reduced model” contains only education, age, sex, race/ethnicity, and calendar year as predictors. The “full model” adds the mediators.

As shown in Supplementary Table 1, the 2011-2018 BRFSS contains a few missing observations. One option for handling missing values is multiple imputation. However, this is not ideal when using the KHB method to parse out the contribution of *specific* mechanisms. Another option is to forgo multiple imputation and conduct the KHB method using the subset of nonmissing observations. This could be problematic if excluding observations biases the model results. We assessed whether this was, in fact, problematic. First, we used the subset of data with nonmissing observations to estimate a model and conduct the KHB method (see the column “Complete Case Dataset” in Table S2). Next, we used all missing and nonmissing observations and the mi command in Stata to build five imputed datasets. Using each dataset, we estimated a model and conducted the KHB method (see the columns “Multiply Imputed Datasets” in Table S2). The results from the models using imputed data were nearly identical to the model using nonmissing data. This shows that using the N=1,716,757 observations with nonmissing data does not bias our results. Note that we do not use standard errors or significance tests in the main analyses, so these supplementary analyses are only concerned with potential bias of the point estimates.

| Abbreviation | Name |  | Abbreviation | Name |
| --- | --- | --- | --- | --- |
| AK | Alaska |  | MT | Montana |
| AL | Alabama |  | NC | North Carolina |
| AR | Arkansas |  | ND | North Dakota |
| AZ | Arizona |  | NE | Nebraska |
| CA | California |  | NH | New Hampshire |
| CO | Colorado |  | NJ | New Jersey |
| CT | Connecticut |  | NM | New Mexico |
| DE | Delaware |  | NV | Nevada |
| FL | Florida |  | NY | New York |
| GA | Georgia |  | OH | Ohio |
| HI | Hawaii |  | OK | Oklahoma |
| IA | Iowa |  | OR | Oregon |
| ID | Idaho |  | PA | Pennsylvania |
| IL | Illinois |  | RI | Rhode Island |
| IN | Indiana |  | SC | South Carolina |
| KS | Kansas |  | SD | South Dakota |
| KY | Kentucky |  | TN | Tennessee |
| LA | Louisiana |  | TX | Texas |
| MA | Massachusetts |  | UT | Utah |
| MD | Maryland |  | VA | Virginia |
| ME | Maine |  | VT | Vermont |
| MI | Michigan |  | WA | Washington |
| MN | Minnesota |  | WI | Wisconsin |
| MO | Missouri |  | WV | West Virginia |
| MS | Mississippi |  | WY | Wyoming |

**Supplementary Table 3 |** U.S. state abbreviations and names

**Supplementary Table 4 |** Percent contribution of nine meditators to the education-health association within U.S. states among adults ages 25-64 from the selected empirical specification (N=1,716,757)

| State | Employment | Income | Smoking | Alcohol | Obese | Married | Children | Coverage | Affordability |
| --- | --- | --- | --- | --- | --- | --- | --- | --- | --- |
| HI | 10.9 | 27.3 | 11.0 | -0.1 | 11.3 | 1.9 | -0.7 | -2.6 | 4.3 |
| SD | 8.8 | 29.5 | 14.0 | -0.3 | 4.2 | 1.3 | 0.2 | -2.1 | 6.7 |
| UT | 7.5 | 26.9 | 14.2 | -0.2 | 8.4 | 1.4 | 3.0 | -4.4 | 8.4 |
| ND | 7.0 | 23.3 | 14.4 | 0.2 | 5.4 | 1.7 | 0.5 | -2.4 | 4.9 |
| IA | 9.3 | 35.9 | 13.7 | -0.6 | 7.9 | 0.6 | 0.3 | -2.5 | 4.7 |
| MT | 9.4 | 31.7 | 10.6 | -0.1 | 5.6 | 1.2 | 0.0 | -3.0 | 5.3 |
| AK | 10.0 | 29.1 | 12.9 | -0.1 | 6.4 | 1.1 | -0.8 | -5.0 | 6.4 |
| MN | 8.5 | 27.6 | 11.5 | -0.2 | 7.9 | 1.4 | 0.3 | -1.3 | 4.4 |
| NE | 9.1 | 31.4 | 12.0 | -0.2 | 5.3 | 0.7 | 0.4 | -2.9 | 7.4 |
| NV | 11.0 | 32.4 | 9.8 | -0.2 | 5.0 | 0.2 | 0.0 | -4.0 | 7.0 |
| ID | 11.0 | 34.9 | 12.2 | -0.2 | 6.1 | 0.0 | 0.9 | -5.5 | 8.6 |
| NJ | 12.3 | 28.8 | 6.9 | -0.2 | 6.5 | 0.9 | 0.2 | -3.1 | 7.4 |
| WY | 10.2 | 26.0 | 11.2 | -0.1 | 7.0 | 1.1 | 0.2 | -3.2 | 6.9 |
| WI | 8.9 | 28.9 | 11.0 | -0.5 | 6.1 | 1.2 | 0.3 | -2.4 | 4.9 |
| CO | 7.5 | 27.0 | 10.7 | 0.0 | 7.3 | 0.8 | 0.0 | -2.7 | 6.5 |
| KS | 11.3 | 33.2 | 12.3 | -0.1 | 5.7 | 0.6 | 0.1 | -4.1 | 7.9 |
| WA | 12.0 | 25.8 | 11.0 | -0.1 | 8.3 | 1.8 | 0.1 | -2.4 | 5.3 |
| NM | 11.7 | 36.1 | 7.3 | 0.0 | 5.3 | 0.1 | -0.4 | -4.1 | 6.3 |
| IL | 10.3 | 28.6 | 8.1 | 0.1 | 6.0 | 1.0 | 0.0 | -1.2 | 5.4 |
| AZ | 12.4 | 33.6 | 8.7 | -0.1 | 5.5 | 0.3 | -0.1 | -3.3 | 6.2 |
| NY | 14.3 | 32.0 | 8.0 | 0.0 | 6.9 | 1.1 | -0.4 | -1.5 | 3.5 |
| LA | 16.5 | 36.4 | 8.2 | 0.0 | 5.1 | 0.1 | -0.3 | -4.4 | 8.6 |
| PA | 13.8 | 33.2 | 9.2 | 0.1 | 7.2 | 0.4 | 0.0 | -4.2 | 5.7 |
| CA | 10.2 | 33.5 | 4.2 | -0.1 | 5.8 | 0.3 | 0.0 | -2.1 | 5.0 |
| TX | 9.8 | 33.5 | 7.0 | -0.1 | 6.1 | 0.7 | -0.6 | -5.6 | 9.8 |
| MD | 13.5 | 25.7 | 8.5 | 0.0 | 6.9 | 1.2 | 0.2 | -2.2 | 5.6 |
| OR | 10.0 | 28.1 | 10.2 | 0.0 | 9.0 | 1.8 | 0.1 | -2.6 | 6.0 |
| MI | 14.0 | 29.5 | 10.4 | -0.3 | 5.5 | 1.3 | 0.1 | -3.2 | 6.1 |
| RI | 16.4 | 32.5 | 8.9 | 0.1 | 5.7 | -0.2 | 0.6 | -1.8 | 5.5 |
| CT | 10.4 | 27.0 | 7.8 | -0.1 | 7.0 | 0.3 | 0.6 | -1.3 | 4.9 |
| MS | 16.9 | 35.7 | 5.6 | 0.0 | 2.7 | 0.5 | 0.0 | -5.5 | 9.0 |
| OK | 15.3 | 36.6 | 11.2 | -0.1 | 5.2 | 0.2 | 0.0 | -4.0 | 8.6 |
| FL | 14.0 | 35.0 | 7.1 | -0.1 | 4.5 | 0.6 | -0.2 | -4.4 | 9.2 |
| IN | 13.8 | 30.3 | 10.5 | 0.0 | 5.3 | 0.9 | 0.2 | -3.8 | 7.3 |
| DE | 12.2 | 30.9 | 7.0 | 0.0 | 5.8 | 1.4 | 0.0 | -2.4 | 5.7 |
| OH | 14.5 | 33.2 | 11.1 | -0.1 | 5.2 | 1.5 | 0.2 | -3.1 | 6.2 |
| SC | 14.9 | 34.3 | 6.8 | 0.0 | 4.3 | 0.6 | -0.1 | -4.8 | 8.9 |
| NH | 13.4 | 30.2 | 9.6 | 0.0 | 6.2 | 0.8 | 0.6 | -3.7 | 5.7 |
| VA | 12.4 | 32.2 | 8.8 | 0.0 | 6.3 | 0.5 | 0.0 | -3.2 | 7.5 |
| ME | 16.4 | 30.1 | 8.6 | 0.0 | 6.8 | 0.5 | 0.6 | -3.8 | 4.1 |
| VT | 12.7 | 25.4 | 8.0 | 0.0 | 8.8 | 1.5 | 0.4 | -1.9 | 3.2 |
| AR | 16.6 | 36.4 | 9.0 | -0.2 | 3.6 | 0.7 | 0.0 | -4.2 | 7.8 |
| MA | 14.3 | 27.8 | 7.9 | 0.0 | 5.7 | 1.2 | 0.3 | -0.9 | 3.2 |
| AL | 17.0 | 35.5 | 6.5 | -0.1 | 3.5 | 0.2 | 0.0 | -4.0 | 8.5 |
| MO | 12.9 | 31.9 | 10.7 | -0.2 | 4.3 | 0.7 | -0.1 | -4.6 | 6.9 |
| KY | 19.0 | 34.0 | 7.6 | 0.0 | 3.8 | 0.3 | -0.1 | -3.7 | 5.6 |
| NC | 11.2 | 36.1 | 6.3 | 0.0 | 5.3 | -0.1 | 0.0 | -4.1 | 8.0 |
| GA | 12.5 | 33.0 | 6.4 | 0.0 | 4.7 | 0.3 | -0.2 | -4.5 | 7.6 |
| TN | 17.1 | 34.5 | 9.3 | 0.1 | 3.7 | 0.5 | -0.2 | -3.0 | 6.7 |
| WV | 18.3 | 32.6 | 7.3 | 0.0 | 3.4 | 0.6 | -0.1 | -3.6 | 6.0 |

*Note:* States are listed in the order that they are shown in Figure 2 in the manuscript.

**
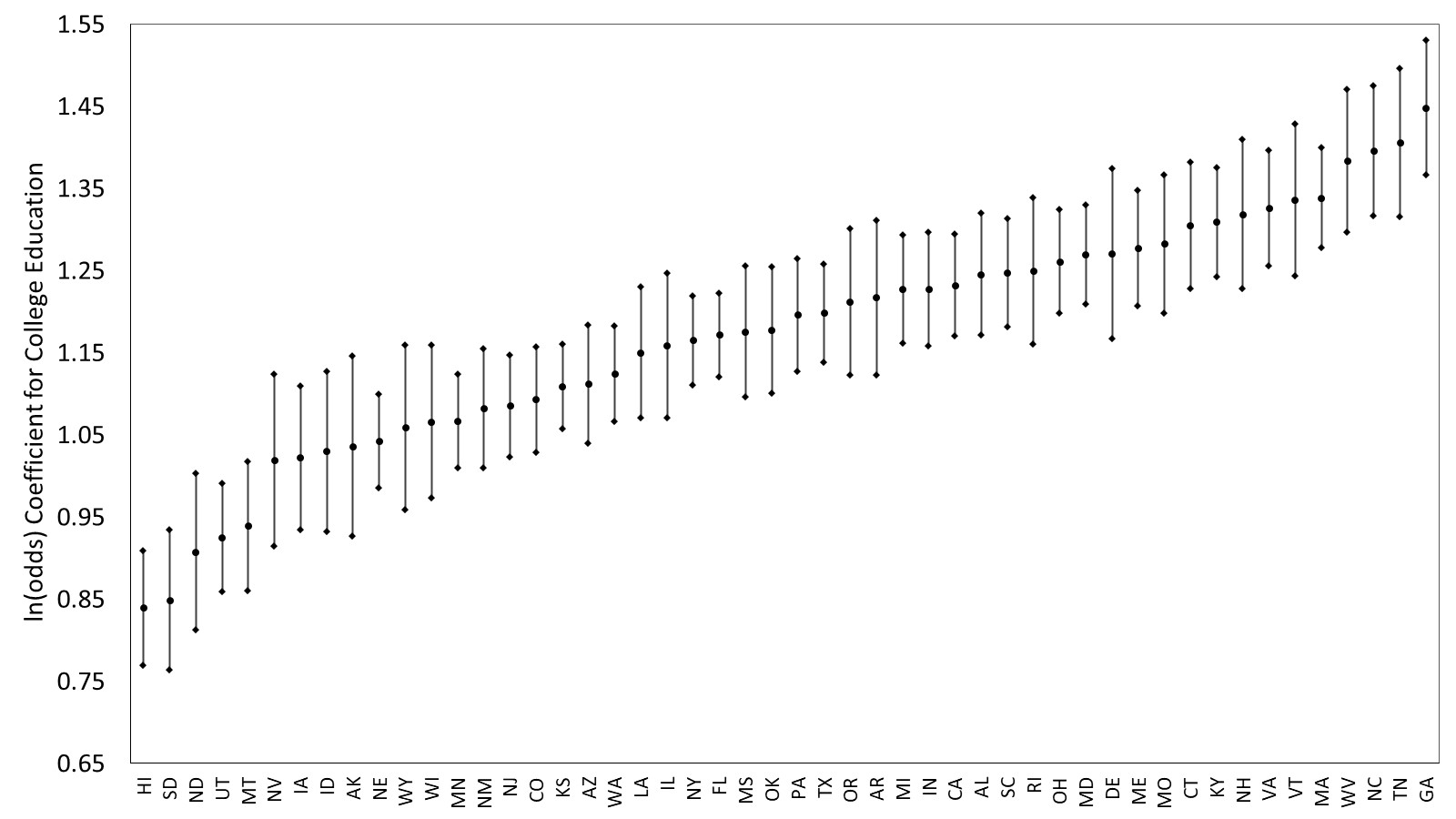
**

**Supplementary Figure 1 |** Point estimates and confidence intervals (CIs) for the college variable, estimated from a separate logistic regression model [ln(odds of favorable health) = college age sex race/ethnicity year] for each state, among adults ages 25–64 years.

*Notes:* The figure uses 95% CIs, useful for assessing whether the point estimate is statistically different from zero.

**
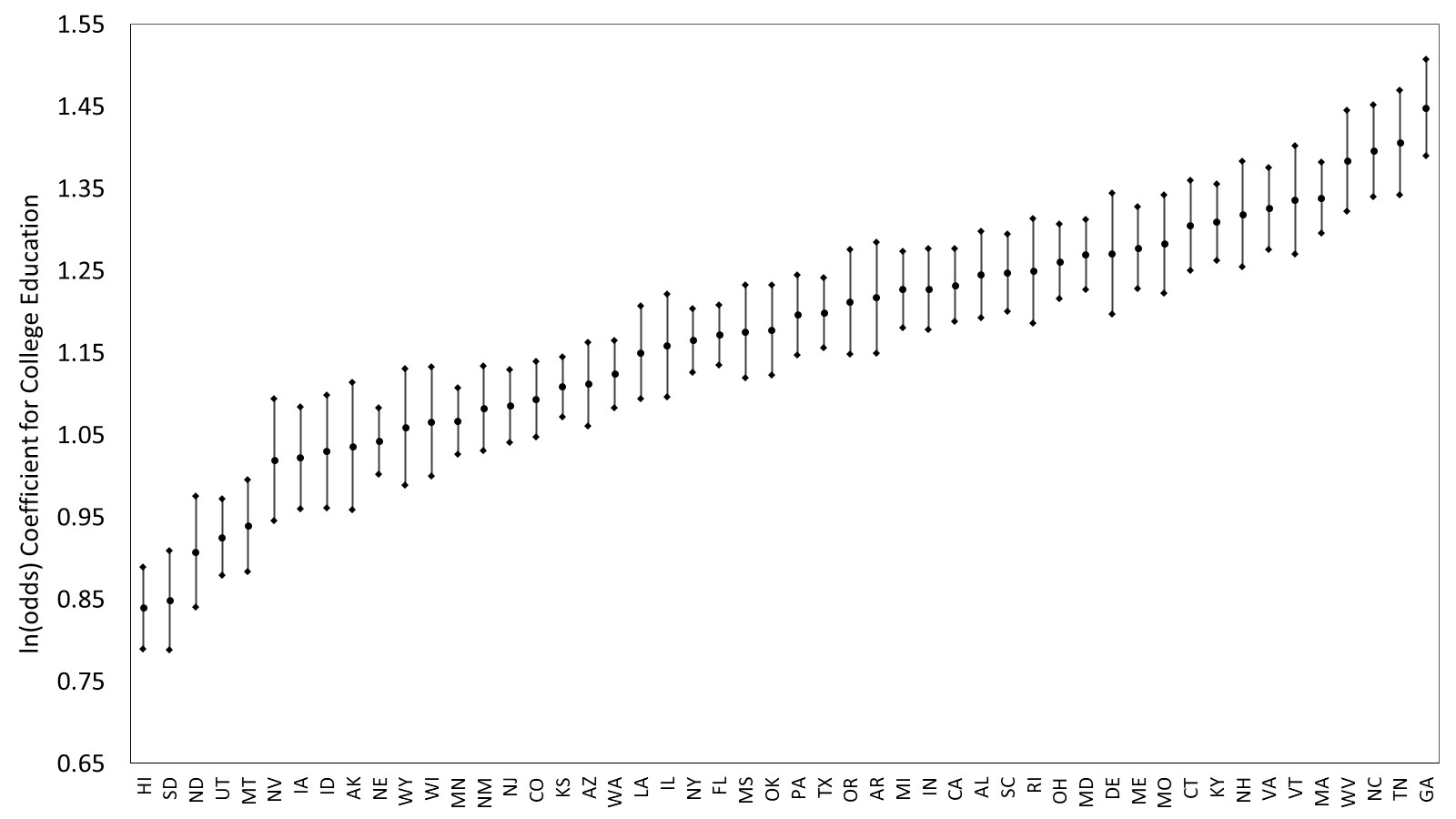
**

**Supplementary Figure 2 |** Point estimate and confidence intervals (CIs) for the college variable, estimated from a separate logistic regression model [ln(odds of favorable health) = college age sex race/ethnicity year] for each state, among adults ages 25–64 years.

*Notes:* The figure uses 83.5% CIs, useful for assessing whether the point estimate for one state is different from that for another state. The 83.5% comes from the adjustment method of Goldstein and Healy (1995) for multiple comparisons while maintaining an average Type I error rate of 0.05.Goldstein H, Healy M Jr. The graphical presentation of a collection of means. Journal of the Royal Statistical Society. Series A. 1995;158(1):175-177.

**
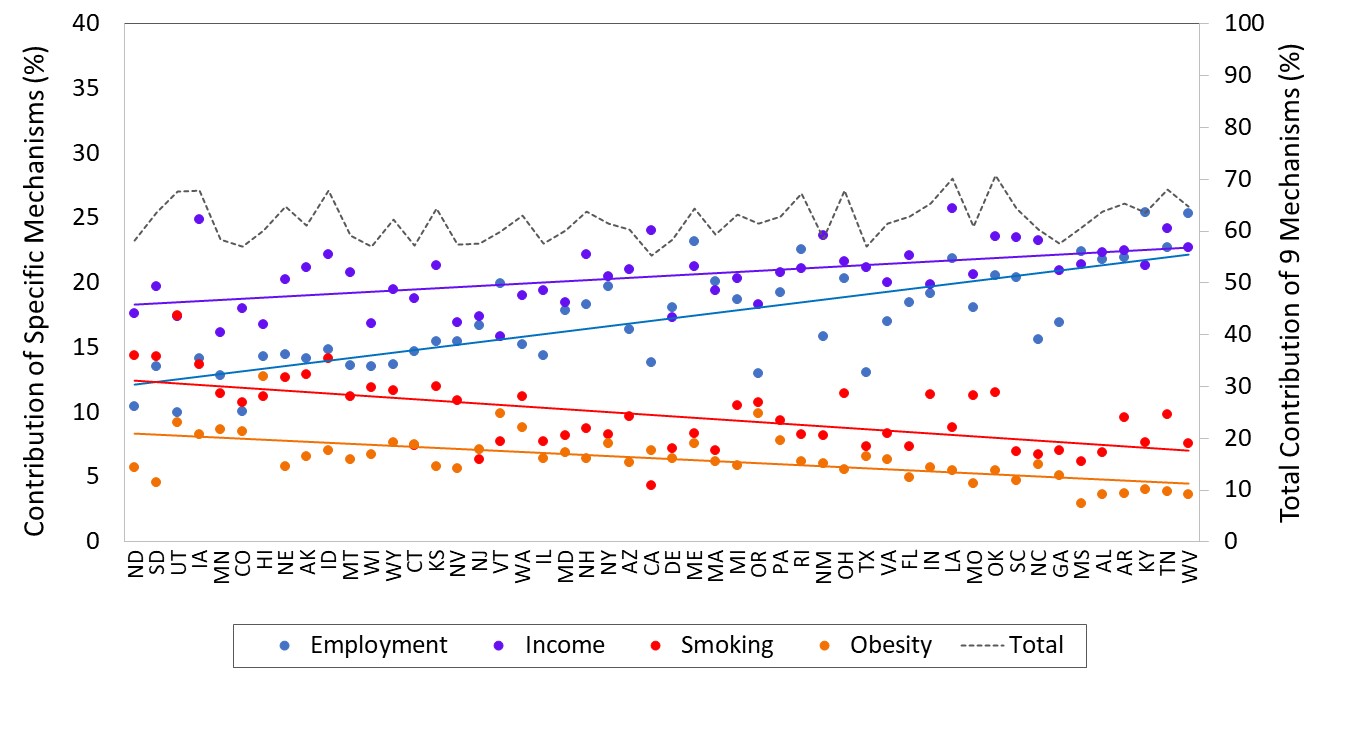

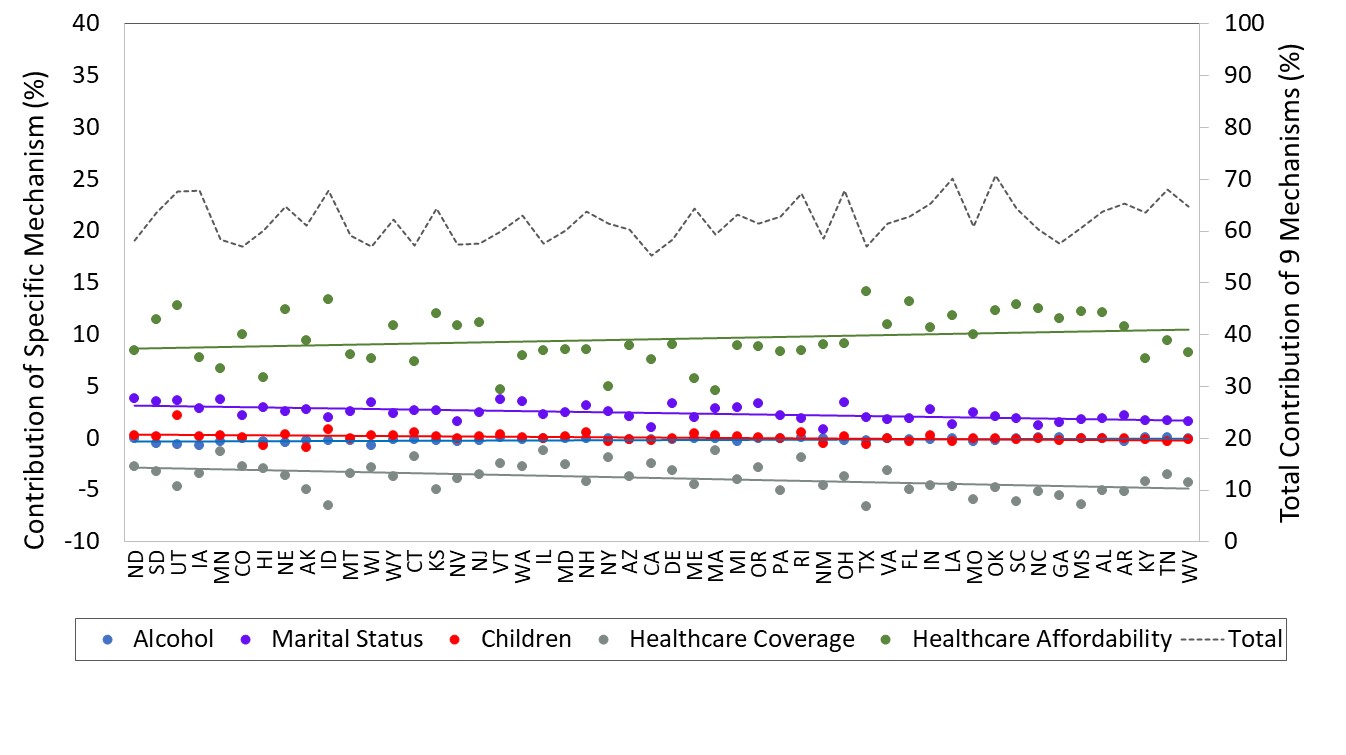
Supplementary Figure 3 |** Contribution of nine mechanisms to the education-health association in U.S. states, using a linear probability model and binary measure of education (college/noncollege).

*Notes:* Data are from the 2011-2018 BRFSS and include adults ages 25-64. States are ordered from left to right in ascending order of the strength of their education-health association, as estimated from a linear probability model with these predictors: college, age, sex, race/ethnicity, and calendar year.

*
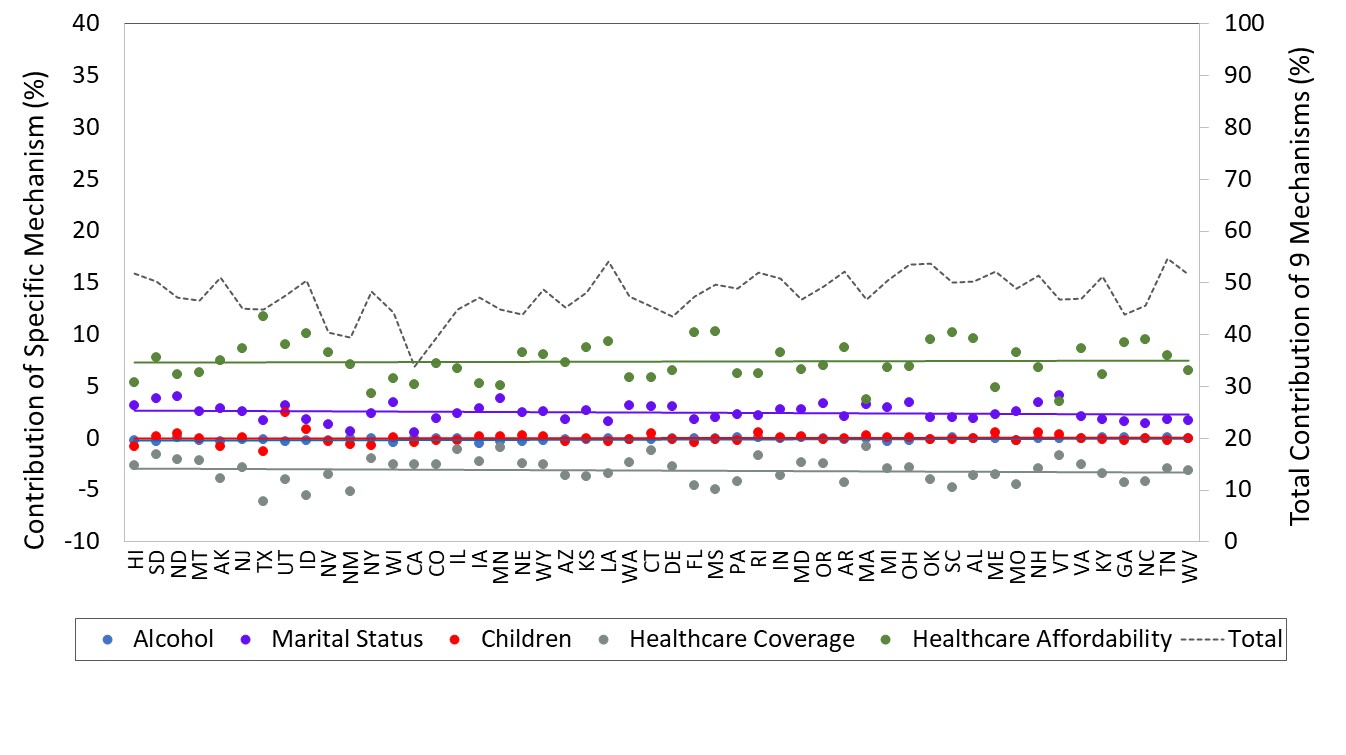
***
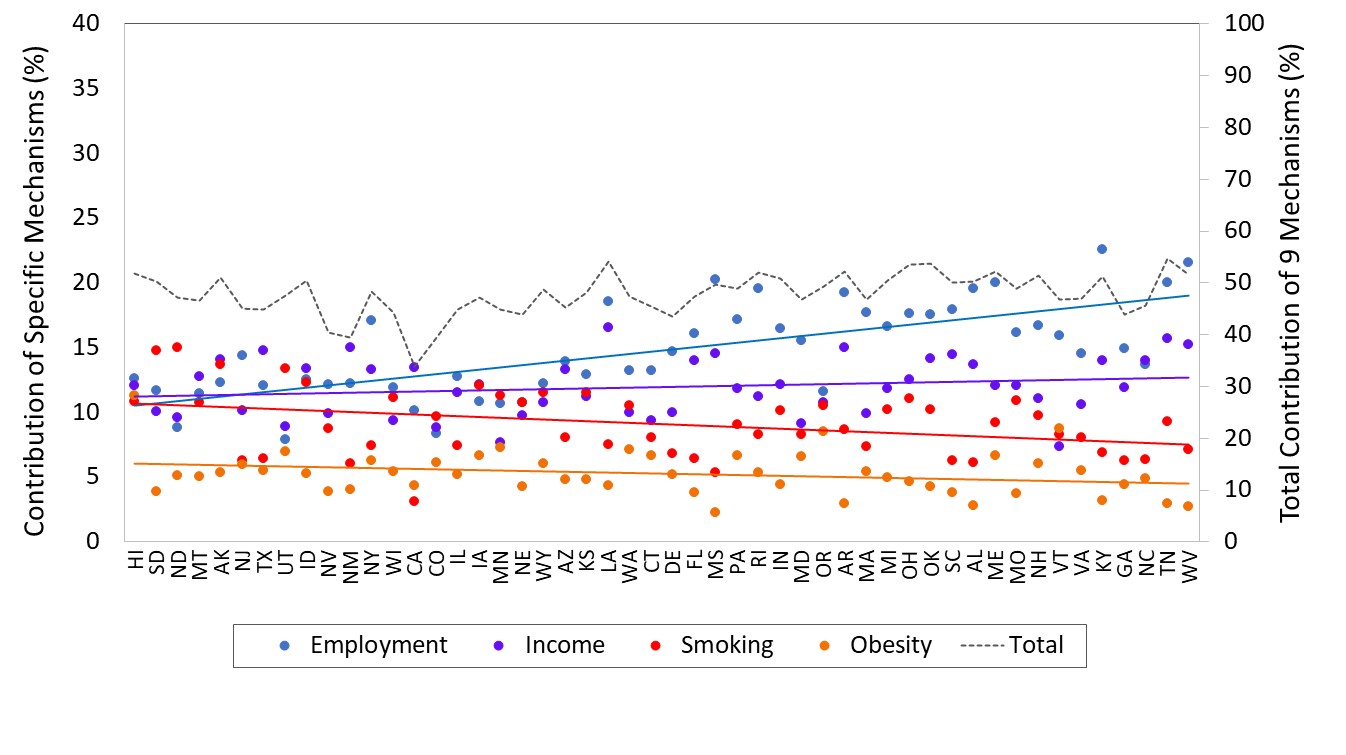
**

**Supplementary Figure 4 |** Contribution of nine mechanisms to the education-health association in U.S. states, using a logit model and continuous measure of education.

*Notes:* Data are from the 2011-2018 BRFSS and include adults ages 25-64. States are ordered from left to right in ascending order of the strength of their education-health association, as estimated from a logit model with these predictors: continuous measure of education, age, sex, race/ethnicity, and calendar year.

**
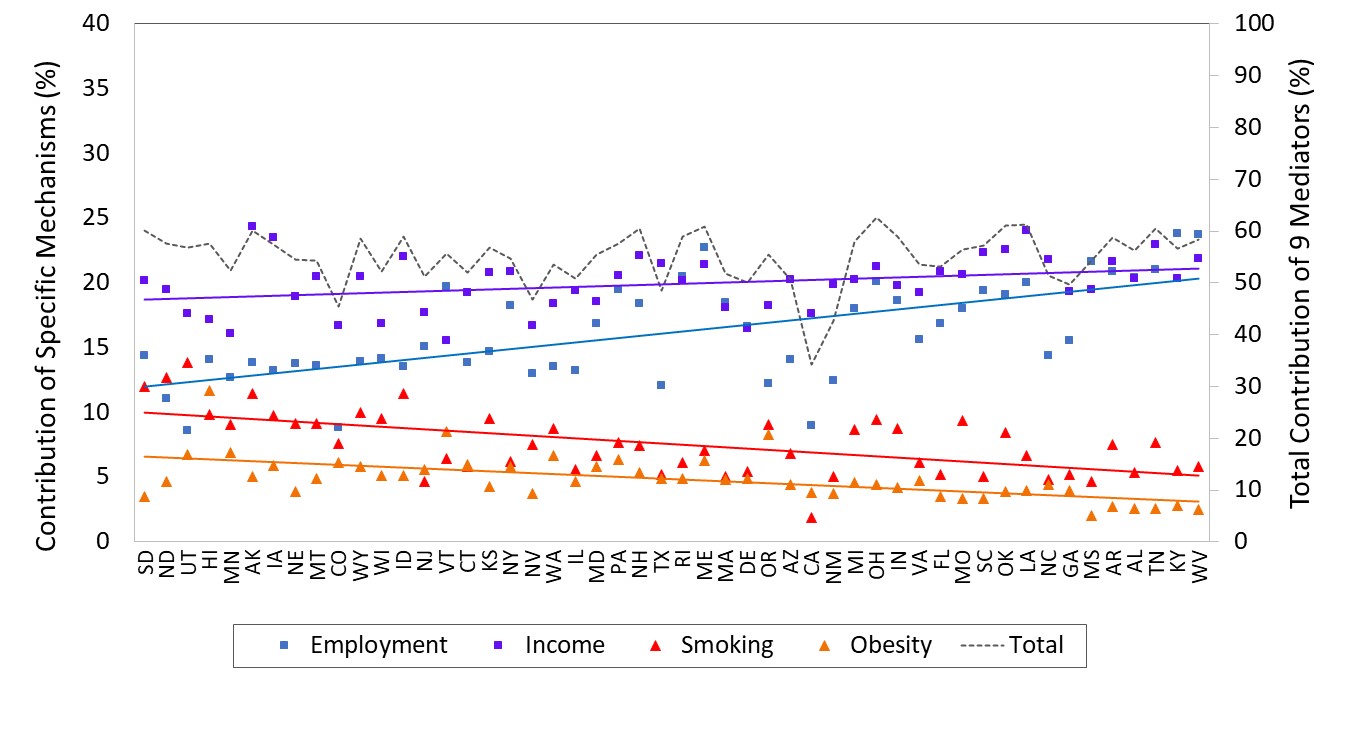

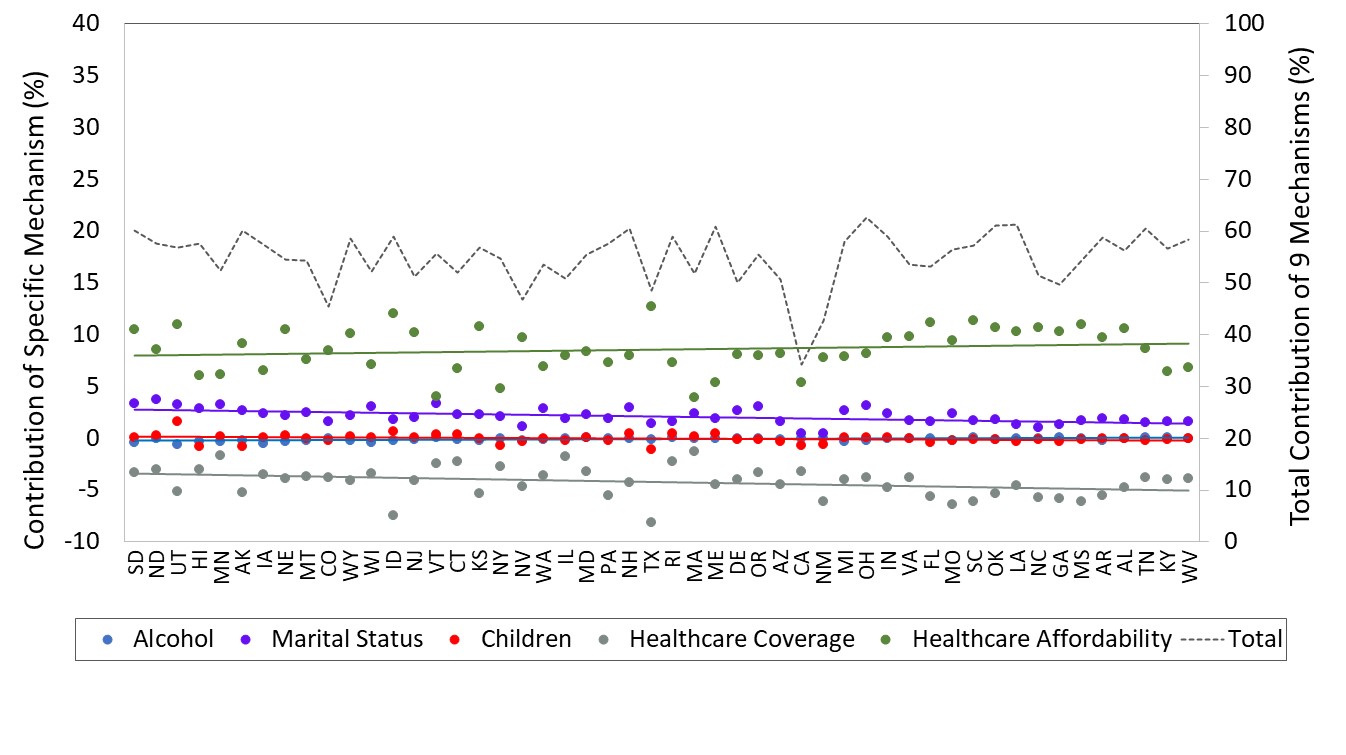
**

**Supplementary Figure 5 |** Contribution of nine mechanisms to the education-health association in U.S. states, using a linear probability model and a continuous measure of education.

*Notes:* Data are from the 2011-2018 BRFSS and include adults ages 25-64. States are ordered from left to right in ascending order of the strength of their education-health association, as estimated from a linear probability model with these predictors: continuous measure of education, age, sex, race/ethnicity, and calendar year.

**
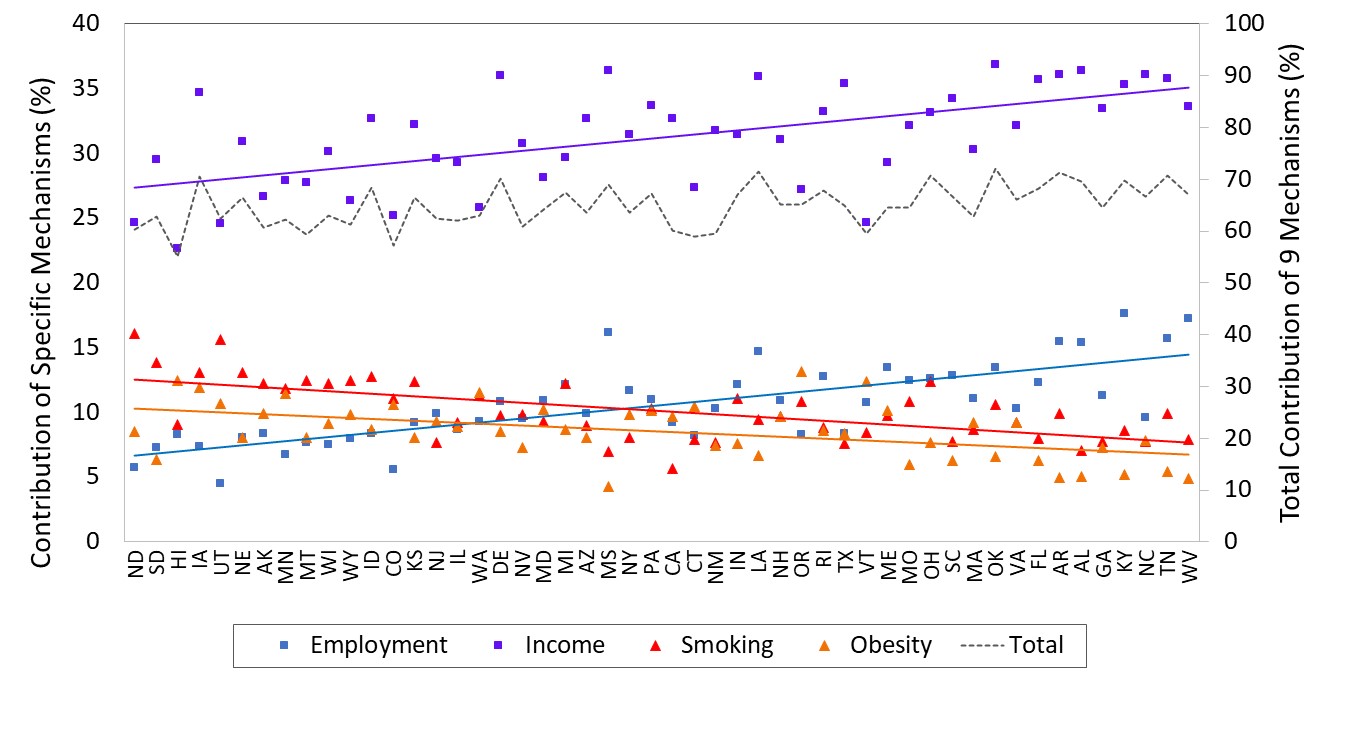

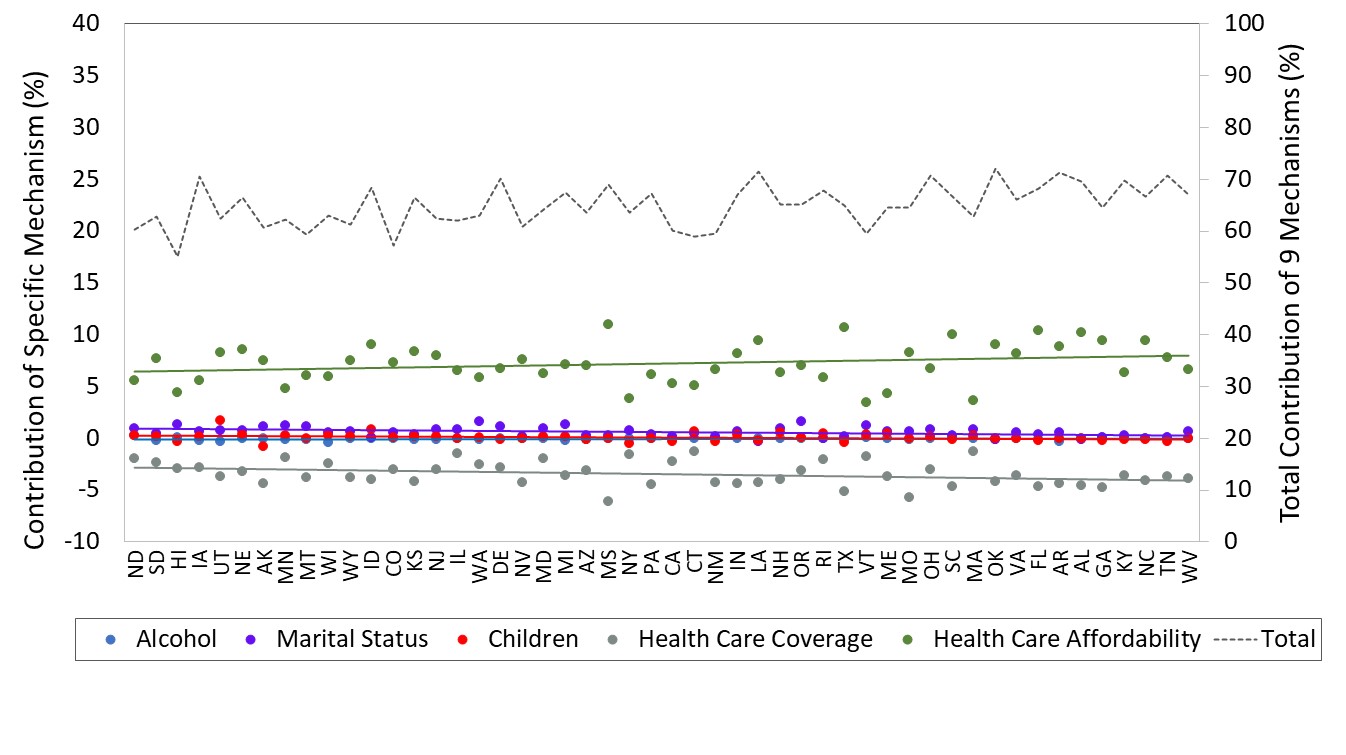
**

**Supplementary Figure 6 |** Contribution of nine mechanisms to the education-health association in U.S. states, using a least squares regression model with self-rated health as a continuous variable and education as a binary variable (college/noncollege).

*Notes:* Data are from the 2011-2018 BRFSS and include adults ages 25-64. States are ordered from left to right in ascending order of the strength of their education-health association, as estimated from an OLS regression model with these predictors: college, age, sex, race/ethnicity, and calendar year.
